# Voltammetric E-Tongue for Honey Adulteration Detection

**DOI:** 10.3390/s21155059

**Published:** 2021-07-26

**Authors:** Paula Ciursa, Mircea Oroian

**Affiliations:** Faculty of Food Engineering, Stefan cel Mare University of Suceava, 720029 Suceava, Romania; paula.ciursa@fia.usv.ro

**Keywords:** voltammetric tongue, honey adulteration, syrups, detection

## Abstract

The aim of this study is to establish the usefulness of an electronic tongue based on cyclic voltammetry e-tongue using five working electrodes (gold, silver, copper, platinum and glass) in honey adulteration detection. Authentic honey samples of different botanical origin (acacia, *tilia*, sunflower, polyfloral and raspberry) were adulterated with agave, maple, inverted sugar, corn and rice syrups in percentages of 5%, 10%, 20% and 50%. The silver and copper electrodes provided the clearest voltammograms, the differences between authentic and adulterated honey samples being highlighted by the maximum current intensity. The electronic tongue results have been correlated with physicochemical parameters (pH, free acidity, hydroxymethylfurfural content—5 HMF and electrical conductivity—EC). Using statistical methods such as Linear discriminant analysis (LDA) and Support vector machines (SVM), an accuracy of 94.87% and 100% respectively was obtained in the calibration step and 89.65% and 100% respectively in the validation step. The PLS-R (Partial Least Squares Regression) model (constructed from the minimum and maximum current intensity obtained for all electrodes) was used in physicochemical parameters prediction; EC reached the highest regression coefficients (0.840 in the calibration step and 0.842 in the validation step, respectively), being followed by pH (0.704 in the calibration step and 0.516 in the validation step, respectively).

## 1. Introduction

Honey is a food product with a high nutritional value known since ancient times for its organoleptic and therapeutic characteristics [1]. Honey must be evaluated because the price is being established based on botanical origin and the presence of adulterants [2]. Among the negative effects of adulterated honey on consumer health are: high blood sugar, weight gain, high blood pressure due to high blood lipids [3]. The honey sector does not require data on the exact degree of adulteration, because the addition of any other substances is prohibited. Therefore, a fast technique is needed to detect the lowest degree of adulteration. Among the most promising techniques that meet these requirements, in addition to being more environmentally friendly than ordinary methods, electronic tongue (e-tongue) has the advantage of being able to be an alternative tool to traditional analytical methods [4]. Electronic tongue can be used in the qualitative [5] but also quantitative characterization of multicomponent [6].

Electronic tongue mimics the taste systems of humans, being developed in many industrial applications due to its simplicity and ruggedness [7]. The efficiency of this sensor depends on the absorption and catalysis of materials into ions. A taste sensor is a low selective sensor that can identify components in a mixture of solutions [8]. Electronic tongue is based on various principles such as electrochemical methods (amperometry, potentiometry, cyclic voltammetry) [9], mass change detection, optical (luminescence, absorbance, reflectance etc.) and enzymatic methods [10,11,12]. A particular importance among the electrochemical methods are the sensors made of simple metals/electrodes with metal oxide, carbon paste electrodes, screen-printed electrodes or graphite-epoxy based electrodes [13], which are immersed in a solution that generates voltage in relation to a potentiometric reference electrode [14]. There are a multitude of sensors designed, but only some have essential characteristics such as selectivity, durability and sensitivity [15].

The component elements of an electronic tongue are: automatic sampler, set of chemical sensors and a software for signal processing and obtaining results [6]. Electronic tongue systems (coupled with chemometric instruments to establish predictive statistical model) are based on a series of non-specific sensors, poorly selective sensors with partial specificity (cross-sensitivity) [16,17]. There are several types of electronic tongue systems, the most widely used being the voltammetric method. It has various advantages such as: high sensitivity, fast detection speed, versatility, simplicity, good signal to noise ratio and robustness [4,18,19]. The principle underlying the operation of the voltampere is the application of a voltage between the working electrode and the reference one by measuring the resulting current between the working electrode and the auxiliary one [20]. The content of total reducing/oxidizing agents present in samples is related to the measurement of the redox potential [21]. Using cyclic voltammetry, the oxidation/reduction peaks are obtained only from their corresponding potential values, being able to identify a certain compound and being obtained also its concentration [22]. The signals collected by the sensors are processed by model recognition tools to generate prediction models that allow the classification and quantification of physicochemical properties of the samples to be analyzed robustness [4].

This method can detect samples of different geographical and botanical origins but can also be used to detect adulterated honey [7]. The use of potentiometric sensors in combination with principal component analysis (PCA) is an alternative in the classification and identification of samples [23]. Other techniques that can be applied are: LDA [24], canonical correlation analysis (CCA) [25], support vector machine (SVM), probabilistic neural network (PNN) and k-nearest neighbour (KNN) [26], discriminant function analysis (DFA) [27], cluster analysis (CA), artificial neural networks (ANN), partial least squares (PLS), principal component regression (PCR) [28].

The purpose of this paper is to show the usefulness of the voltammetric tongue consisting of five working electrodes in correlation with the physicochemical parameters in the detection of adulterated honey samples with different types of syrups (corn, rice, inverted sugar, agave and maple).

## 2. Materials and Methods

### 2.1. Materials

Seventy authentic honey samples of different botanical origin (acacia—22 samples, tilia—22 samples, sunflower—22 samples, polyfloral—2 samples, raspberry—2 samples) were purchased from local beekeepers from Suceava County, Romania. A sample of each variety of honey was adulterated by adding syrup such as rice, corn, inverted sugar, agave and maple in different percentages (5%, 10%, 20% and 50%), thus, resulting 105 adulterated honey samples (including the five adulteration agents as: rice 5%—5 samples, rice 10%—5 samples, rice 20%—5 samples, rice syrup 50%—5 samples, rice syrup 100%—1 sample, corn syrup 5%—5 samples, corn syrup 10%—5 samples, corn syrup 20%—5 samples, corn syrup 50%—5 samples, corn syrup 100%—1 sample, inverted sugar syrup 5%—5 samples, inverted sugar syrup 10%—5 samples, inverted sugar syrup 20%—5 samples, inverted sugar syrup 50%—5 samples, inverted sugar syrup 100%—1 sample, maple syrup 5%—5 samples, maple syrup 10%—5 samples, maple syrup 20%—5 samples, maple syrup 50%—5 samples, maple syrup 100%—1 sample, agave syrup 5%—5 samples, agave syrup 10%—5 samples, agave syrup 20%—5 samples, agave syrup 50%—5 samples, agave syrup 100%—1 sample, respectively). Only the inverted sugar syrup was obtained in the laboratory using sucrose (the solution being corrected by adding citric acid), the rest of the syrups were purchased from the commercial market.

### 2.2. Methods

#### 2.2.1. Physicochemical Parameters

##### pH and Free Acidity

The pH and free acidity of 10 g of honey dissolved in 75 mL of distilled water were determined using the Titroline device (SCHOTT Instrument, Germany). pH measurement was performed in solution and the free acidity was determined by titration with 0.1 M sodium hydroxide solution to pH 8.30. The results were expressed in milliequivalents/kg of honey, the calculation being performed using the following formula:

Free acidity = mL of 0.1 M NaOH × 10(1)

##### Electrical Conductivity (EC)

The EC of a solution of 20 g of honey dissolved in 100 mL of distilled water was measured using an XL 30 conductometer (Fisher Scientific, Schwerte, Germany). The results obtained were expressed in microSiemens per centimeter (μS·cm^−1^).

##### Hydroxymethylfurfural (HMF) Content

The hydroxymethylfurfural content was determined by the spectrophotometric method proposed by White [29]. An amount of 5 g of honey was dissolved in 25 mL of ultrapure water. The solution was transferred to a 50 mL volumetric flask, over which 0.5 mL of Carrez I solution was added and mixed. Then, 0.5 mL of Carrez II solution was added and made up with ultrapure water. The solution obtained was filtered and the first 10 mL were rejected. In two tubes, 5 mL of filtrate were introduced over which 5 mL of ultrapure water (in the first test tube) and 5 mL of 0.2% sodium bisulphite were added (in the second test tube representing the reference solution). The absorbance of the sample solution against the reference solution at 284 and 336 was read using a UV-3600 spectrophotometer (Schimadzu Corporation, Japan). The HMF content was calculated using the following formula, the result being expressed in mg/kg:

HMF = (A_284_ − A_336_) × 149.7 × 5 × D/W, (2)

where: A_284_—absorbance at 284 nm, A_336_—absorbance at 336 nm, D—dilution factor, W—weight of the honey sample (g).

#### 2.2.2. Electrochemical Measurement

The solution to be analyzed was prepared by dissolving 8 g of sample, followed by transfer into a 50 mL volumetric flask and filled to the mark with ultrapure water. The measuring system used was a PGSTAT 204 with FRA32M module (Metrohm, Filderstadt, Germany) using a voltametric cell composed of: reference electrode (Ag/AgCl), counter electrode (platinum) and working electrode (glass carbon, gold—Au, silver—Ag, platinum—Pt and cooper—Cu). The electrodes were purchased from Methrom (Filderstadt, Germany), the counter electrode and four working electrodes (glass carbon, gold, silver and platinum) had 2 mm in diameter and the copper electrode had 5 mm. The analysis was made using the cyclic voltammetry ranging the voltage between −1 V to +1V to −1 V. Prior to the analysis, the solution was placed into the electrochemical cell and the electrodes were immersed for 5 min, for achieving the electrochemical equilibria, and after it was done the cyclic voltammetry analysis. The scanning rate was set at 0.5 mV/s. Between two analysis the electrodes were rinsed with ultrapure water and sanded with filter paper. The data were recorded and analyzed with NOVA 2.0 software (Metrohm, Filderstadt, Germany). All analyzes were performed in triplicate.

### 2.3. Statistical Analysis

The results were submitted to analysis of variance (ANOVA) using XLSTAT trial version (Microsoft, Charlotte, NC, USA). Fisher’s least significant difference (LSD) procedure was used at the 95% confidence level. The LDA and SVM were made using XLSTAT trial version (Microsoft, Charlotte, NC, USA).

## 3. Results and Discussion

### 3.1. Influence of Honey Adulteration on Physicochemical Parameters

The results of the physicochemical parameters obtained for the authentic and adulterated honey are presented in Table 1. For the free acidity and for the HMF content the differences were significant (*p* < 0.001), while for the EC and pH were not significant (*p* > 0.05).

Organic acids, especially gluconic acid, in equilibrium with lactones or esters and some inorganic ions, confer the acidity of honey [30]. Fermentation of sugars with alcohol formation, produced under the action of microorganisms, followed by oxidation and formation of carboxylic acids lead to a high free acidity [31]. Adulterated honey samples showed a significant decrease (*p* < 0.001) in free acidity value from 28.14 meq/kg in authentic honey to 11.69 meq/kg. Not only direct adulteration by adding syrups can cause a decrease in free acidity values, but also, indirect adulteration, by intensive feeding of bees with sugar syrups. Özcan et al. [32] observed this change in free acidity in honey resulting from feeding bees with two types of syrups: saccharose and inverted saccharose. The lowest value was in the case of inverted saccharose syrup, being explained by the higher content of dissociated organic acids.

Naturally, the pH of honey is acidic, due to the presence of organic acids. They contribute both to the stability and flavor of honey [33]. The pH value of the adulterated samples showed a negligible decrease. A decrease of pH value was also observed in the case of adulterated honey with hydrolyzed inulin syrup, malt wort, glucose [21] and glucose-fructose syrup [34]. In addition, adulterated honey samples with a 4% starch solution and molasses showed a decrease of 10.1% in pH value compared to pure honey [35].

The EC depends on the content of proteins and mineral salts, providing information about the botanical origin of honey [36]. The EC can be considered the fastest method in the routine control of honey [37] and also a good criterion in evaluating the purity of honey but also its botanical origin [38]. Adulterated honey presented a decrease in EC values, which can be explained by the low presence of the concentration of mineral salts, proteins and organic acids from commercial syrups composition compared to authentic honey. Bodor et al. [34], also, obtained a decrease in EC in the case of linden and acacia honeys adulterated with a glucose-fructose syrup. Lower values were also determined in adulterated honey samples with glucose, molasses and 4% starch solution [35].

Fresh honey has small amounts or even traces of HMF, the formation being slow as long as the storage period and temperature are appropriate. Temperature exposure as well as honey adulterated with inverted sugar syrup lead to the formation of a high amount of HMF [39]. The honey adulteration led to a significant increase in HMF content (7 times higher), exceeding the maximum allowed limit of 40 mg/kg. The high HMF content in adulterated honey samples was also observed in other studies [40,41]. As in the case of free acidity, the HMF content increases significantly in honey produced by feeding bees with inverted saccharose syrup, being attributed to the heating process to which the syrup was subjected to obtain [32].

### 3.2. Voltammetric Tongue

**Figure 1:**
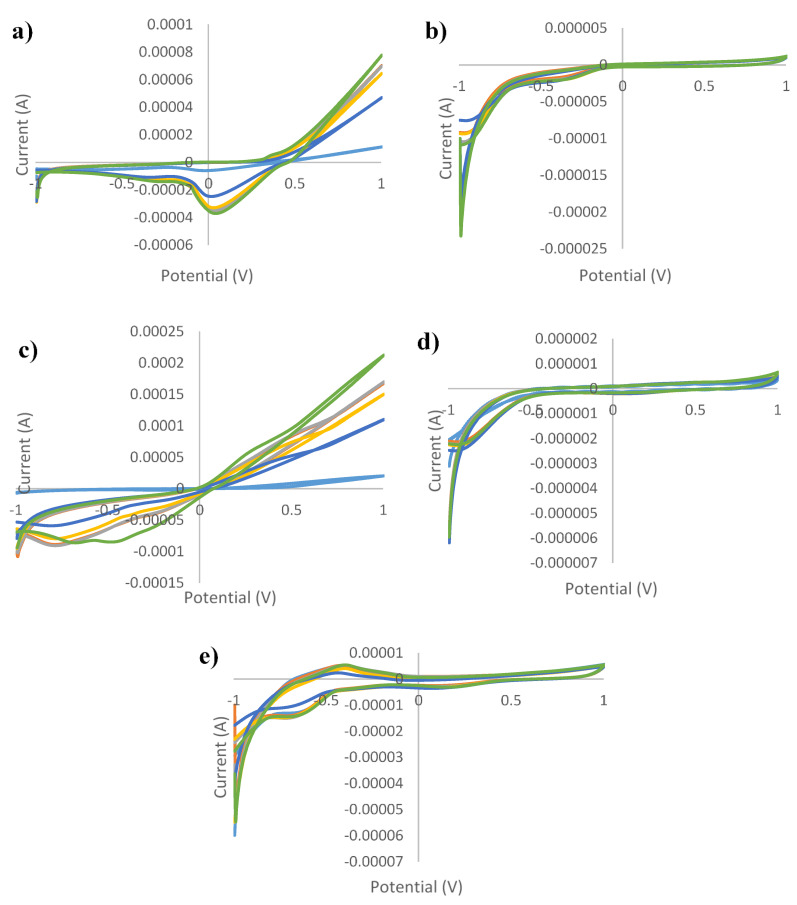
Cyclic voltammograms of authentic and adulterated (with agave syrup) *tilia* honey solutions for: (**a**) silver electrode; (**b**) gold electrode; (**c**) copper electrode; (**d**) glass electrode; (**e**) platinum electrode and green line—authentic honey; light blue line—agave syrup; red line—honey adulteration 5%; gray line—honey adulteration 10%; orange line—honey adulteration 20%; dark blue—honey adulteration 50%.

**Figure 2:**
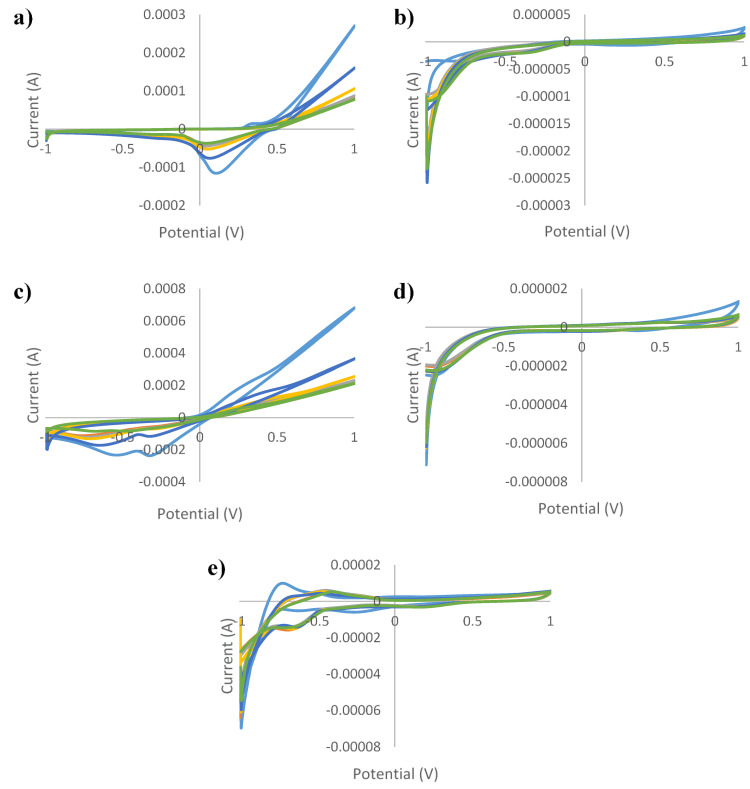
Cyclic voltammograms of authentic and adulterated (with maple syrup) *tilia* honey solutions for: (**a**) silver electrode; (**b**) gold electrode; (**c**) copper electrode; (**d**) glass electrode; (**e**) platinum electrode and green line—authentic honey; light blue line—agave syrup; red line—honey adulteration 5%; gray line—honey adulteration 10%; orange line—honey adulteration 20%; dark blue—honey adulteration 50%.

**Figure 3:**
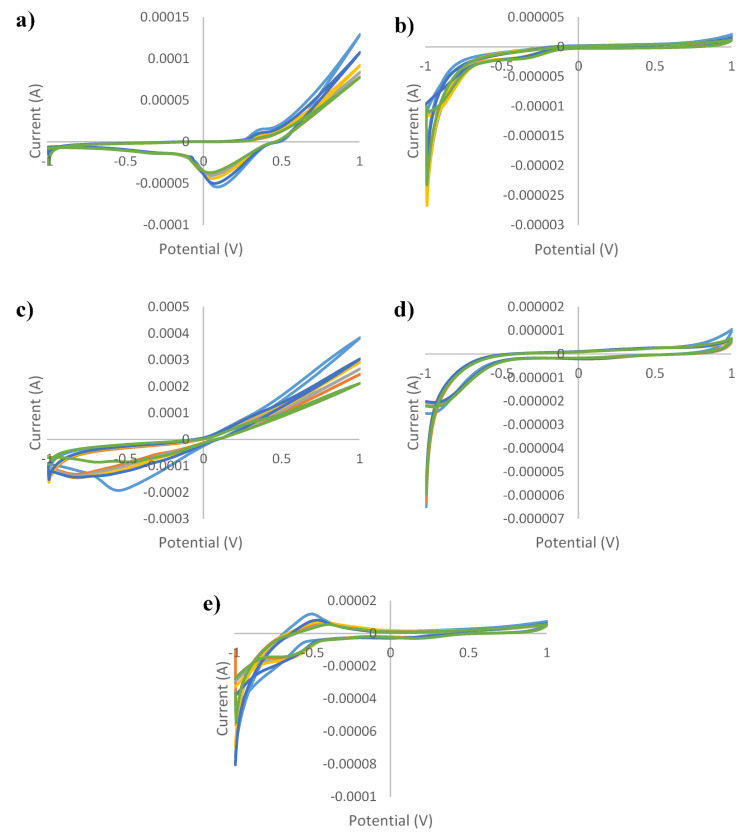
Cyclic voltammograms of authentic and adulterated (with rice syrup) *tilia* honey solutions for: (**a**) silver electrode; (**b**) gold electrode; (**c**) copper electrode; (**d**) glass electrode; (**e**) platinum electrode and green line—authentic honey; light blue line—agave syrup; red line—honey adulteration 5%; gray line—honey adulteration 10%; orange line—honey adulteration 20%; dark blue—honey adulteration 50%.

**Figure 4:**
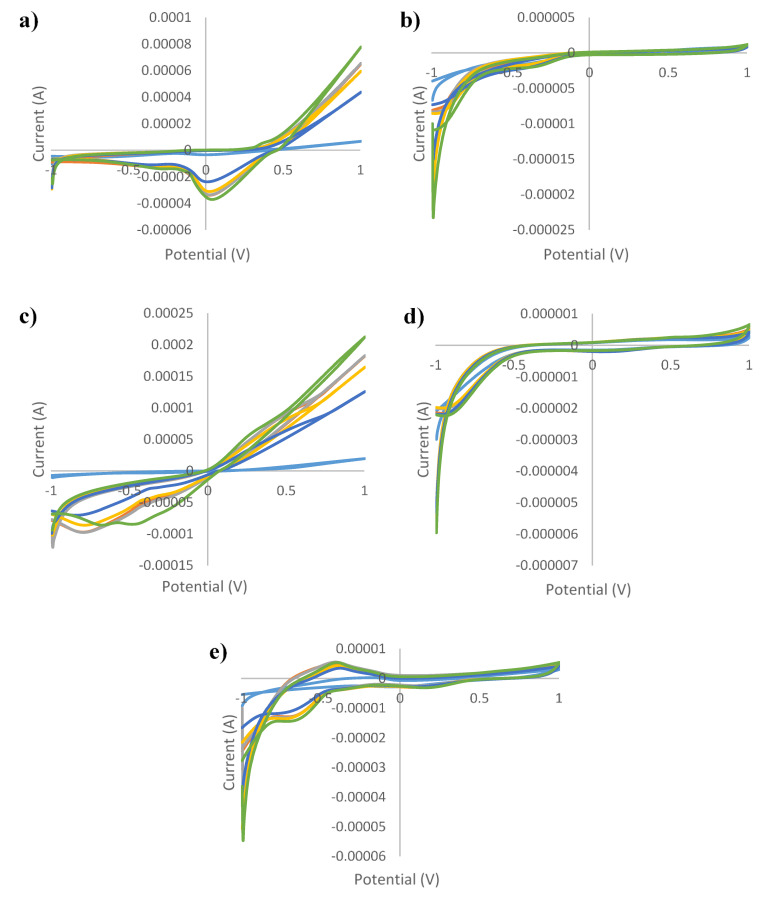
Cyclic voltammograms of authentic and adulterated (with corn syrup) tilia honey solutions for: (**a**) silver electrode; (**b**) gold electrode; (**c**) copper electrode; (**d**) glass electrode; (**e**) platinum electrode and green line—authentic honey; light blue line—agave syrup; red line—honey adulteration 5%; gray line—honey adulteration 10%; orange line—honey adulteration 20%; dark blue—honey adulteration 50%.

**Figure 5:**
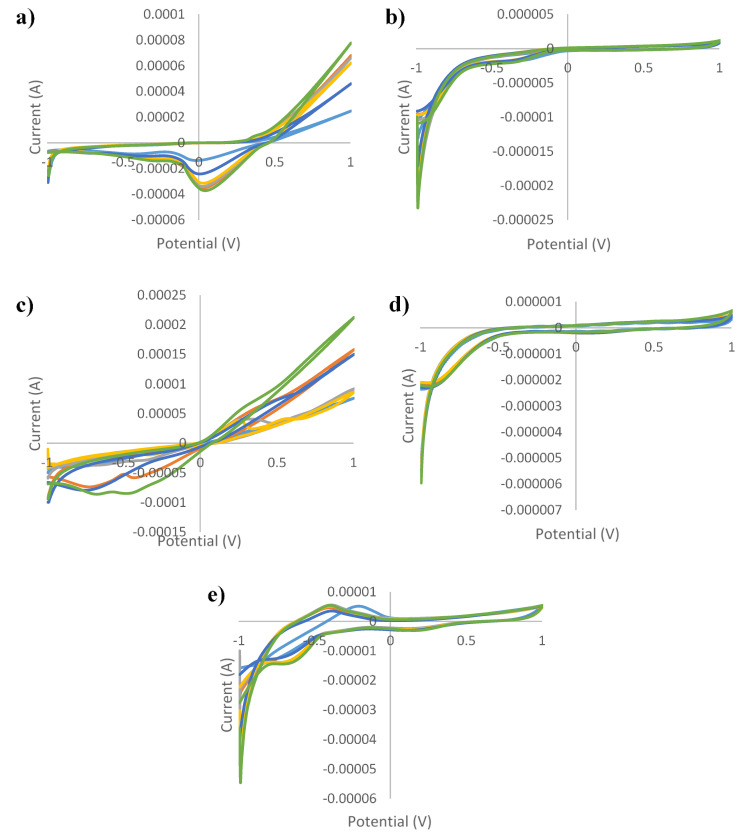
Cyclic voltammograms of authentic and adulterated (with inverted sugar syrup) tilia honey solutions for: (**a**) silver electrode; (**b**) gold electrode; (**c**) copper electrode; (**d**) glass electrode; (**e**) platinum electrode and green line—authentic honey; light blue line—agave syrup; red line—honey adulteration 5%; gray line—honey adulteration 10%; orange line—honey adulteration 20%; dark blue—honey adulteration 50%.

Figure 1, Figure 2, Figure 3, Figure 4 and Figure 5 show the cyclic voltammograms of authentic and adulterated honey solutions. The current variations depending on the adulteration agent/degree of adulteration can be observed most clearly when Ag and Cu were used as working electrodes. By applying a voltage of 1 V, the differences between the authentic sample, the syrup and adulterated samples was distinguished. The highest current intensity value for the authentic sample was recorded when the Cu electrode was used (0.2089 mA) and the lowest was for the glass electrode (0.0006 mA). In the case of syrups, also, for the Cu electrode the highest values were observed (0.6715 mA—maple syrup followed by 0.3777 mA—rice syrup) and the lowest were for the glass electrode (0.0002 mA—corn syrup, 0.0003 mA—agave and inverted sugar syrups). The honey adulteration led to significant changes in the value of the current intensity. For the Ag electrode, the adulteration honey samples with 5% maple and rice syrups showed values of current intensity of 0.0781 mA and 0.0764 mA, close to those of authentic honey (0.0762 mA). Also, the addition of the same syrups in a percentage of 50% produced an increase to 0.1571 mA (in honey adulterated with maple syrup) and 0.1055 mA (honey adulterated with rice syrup), while the other syrups produced a decrease (0.0460 mA, 0.0450 mA and 0.0429 mA for adulterated samples with 50% agave, inverted sugar and corn syrups, respectively). For the Au electrode, the maple and rice syrups added in a percentage of 50% led to an increase from 0.0011 mA in the authentic honey, to 0.0014 mA in adulterated honey for both types of syrups, and in the case of the other types of syrups occurred a decrease to 0.0008 mA in honey adulterated with 50% corn syrup. Regarding the Cu electrode, the increase was from 0.2089 mA (authentic honey) to 0.3589 mA (in honey adulterated with 50% maple syrup), respectively 0.2984 mA (in honey adulterated with 50% rice syrup), and the most significant decrease occurred in honey adulterated with 50% agave syrup (0.1082 mA). The current intensity value for honey adulterated with 5% maple syrup (0.2107 mA) was the closest to the value of the authentic honey; the increase in the percentage of added syrup led to an increase in current intensity. The glass electrode recorded the lowest values, the honey adulterated with corn syrup having a value of 0.0003 mA, as opposed to 0.0006 mA, in the case of authentic honey. Last but not least, for the Pt electrode were observed fluctuations depending on the adulteration agent added, increasing from 0.0053 mA (in authentic honey) to 0.0056 mA, 0.0058 mA and 0.0060 mA for honey adulterated with 50% maple, rice and corn syrups, respectively. The addition of 50% inverted sugar syrup led to a significant decrease (0.0041 mA). Although, by using Cu and Ag electrodes, adulterated samples could be differentiated by visualizing voltammograms, the other electrodes were also useful in providing data that were used for statistical analysis. In addition, not only the maximum of current intensity was studied but also its minimum. The differences cannot be seen with the naked eye, because there are overlaps. For example, using the Pt electrode there were differences by applying a voltage of −1V. Thus, the addition of 50% agave, corn and inverted sugar syrups led to a decrease in current intensity from −0.0168 mA in authentic honey to values of −0.0115 mA, −0.01146 mA and −0.0130 mA, while the other types of syrups produced an increase: −0.0228 mA (adulterated honey with 50% rice syrup) and −0.0310 mA (adulterated honey with 50% maple syrup).

Electronic tongue based on voltammetry has proven to be an effective method in detecting adulterated honey samples, similar results are being reported by other researchers. The principal component analysis and electronic tongue data obtained from Au, Ag, Pt and glass electrodes managed to differentiate the samples of pure honey from those adulterated with glucose syrup and saccharose from a degree of adulteration of 2% [7]. Also, the electronic tongue consisting of the same four working electrodes and the physicochemical properties reached a 97.50% correct classification of pure and adulterated honey [21]. Sobrino-Gregorio et al. [4] used as working electrodes Ir, Rh, Pt and Au to detect adulterated honey with corn syrup, rice and barley. Statistical analyzes such as PCA and PLS were used successfully, the best results being obtained for sunflower honey adulterated with corn syrup (r = 0.997), the same value being obtained for heather honey adulterated with barley syrup. In addition, Guellis et al. [42] used cyclic voltammetry (Cu/CuO electrode) and UV—vis spectrophotometry with statistical methods (PCA and HCA—hierarchical cluster analysis) succeeding in discriminating pure honey and syrup samples but also those adulterated in different percentages. In another study, Bodor et al. [34] evaluated the sensory profile of authentic and adulterated acacia honey with sugar syrup in percentages of 10%, 20% and 50%. Adulterated honey with 10% and 50% syrup had low scores for sweet and floral taste and adulterated honey with 20% and 50% had high scores for caramel taste compared to authentic honey.

### 3.3. Honey Classification Using Statistical Analysis

The honey classification (authentic vs. adulterated) was realized using the linear discriminant analysis and support vector machine in three different ways: a. e-tongue data (minimum and maximum current observed for all the five working electrodes), b. physicochemical parameters (free acidity, pH, 5-HMF and EC) and c. physicochemical parameters and e-tongue data.

In order to evaluate the performance of each statistical method, it were calculated the accuracy, sensitivity (measures the proportion of true positives that are correctly identified) and specificity (measures the proportion of true negatives that are correctly identified) as [43]:

$$\text{Accuracy}=\frac{\text{True}\;\text{positives}\;+\;\text{True}\;\text{negatives}}{\text{True}\;\text{positives}\;+\;\text{True}\;\text{negatives}\;+\;\text{False}\;\text{positives}\;+\;\text{False}\;\text{negatives}}\;\text{Sensitivity}=\frac{\text{True}\;\text{positives}}{\text{True}\;\text{positives}+\text{False}\;\text{negatives}}\;\text{Specificity}=\frac{\text{True}\;\text{negatives}}{\text{True}\;\text{negatives}+\text{False}\;\text{positives}}$$

#### 3.3.1. Linear Discriminant Analysis

The LDA models was used to discriminate the authentic samples from the adulterated ones (honey adulterated with inverted sugar, agave syrup, corn syrup, rice syrup and maple syrup); the model is decomposing the three methods presented above (e-tongue data, physicochemical parameters and e-tongue + physicochemical parameters) to the statistical analysis and the sample category in the same time for the extraction of effective information which can be used for the classification. The accuracy, sensitivity and specificity of the models generated by using e-tongue data with LDA are reported in Table 2. The LDA accuracy (the model constructed with e-tongue data) was 92.31% for the calibration and 96.55% for the validation step, the sensitivity was 82.61% for the calibration and 91.67% for the validation step, while the specificity was 98.59% for the calibration and 100.00% for the validation step. From the 71 adulterated samples 70 were correctly classified in the calibration step, while in the validation all the 34 adulterated samples were correctly classified. In the calibration step from the 46 pure honeys, 8 were not classified correctly, while in the validation only 2 samples from 24 were classified as adulterated ones. In the case of physicochemical parameters the LDA accuracy and sensitivity was lower than in the case of e-tongue, while when it was used the physicochemical parameters and e-tongue data showed that only the accuracy and sensitivity of the calibration steps were higher than when the e-tongue data were used for LDA.

#### 3.3.2. Support Vector Machines Model

SVM is an algorithm used for the classification in binary code and is based on a procedure that finds a special type of linear model called the maximum-margin hyper-plane. In this study, the linear kernel algorithm was used for the separation of the two groups studied (authentic/adulterated) [44]. The statistical parameters of the SVM model based a. e-tongue data (minimum and maximum current observed for all the five working electrodes), b. physicochemical parameters (free acidity, pH, 5-HMF and EC) and c. physicochemical parameters and e-tongue data are presented in Table 2. As it can be observed all the samples were classified correctly (pure and adulterated ones).

### 3.4. Partial Least Squares Regression Correlation of e-Tongue Data with Physicochemical Parameters

**Figure 6:**
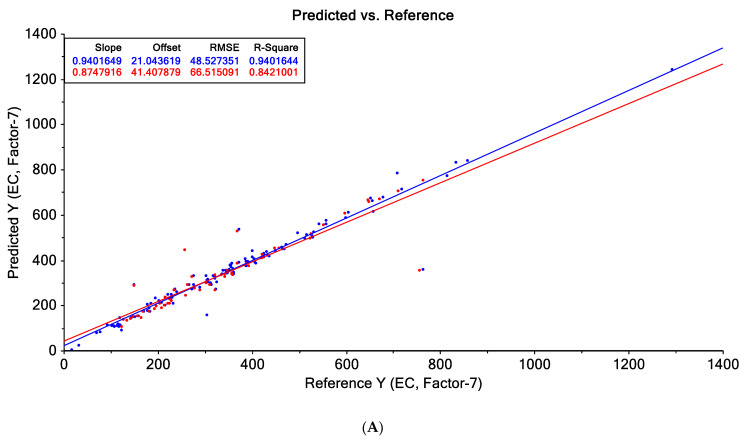

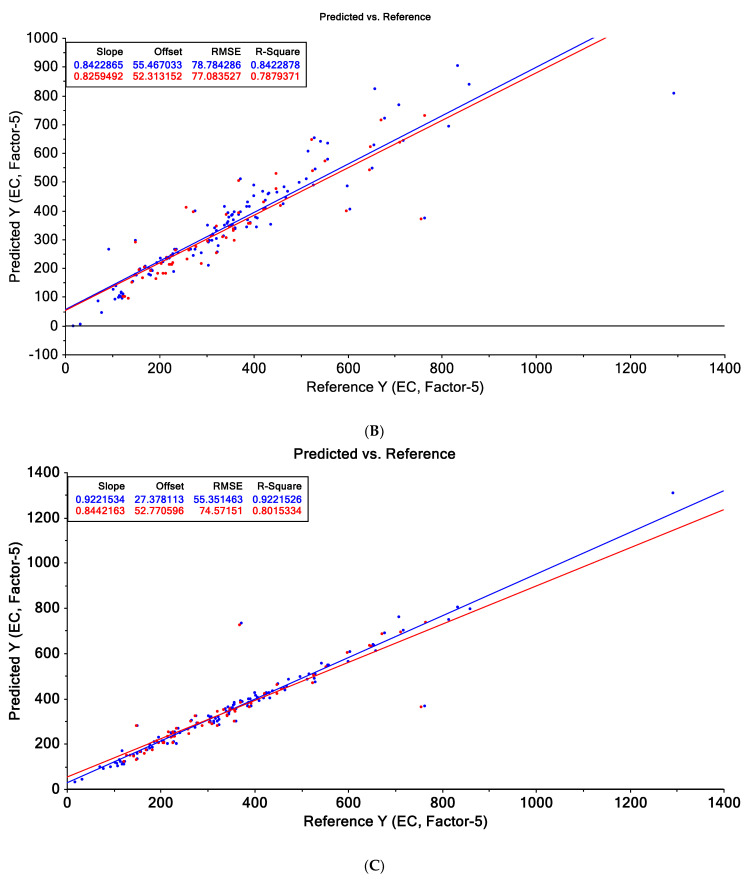

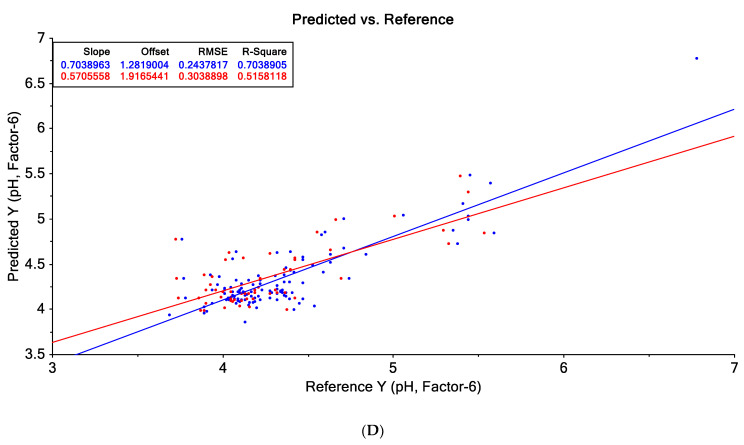
Experimental vs. predicted data using PLSR for: (**A**). EC prediction using minimum and maximum intensity of the current obtained for all the electrodes, (**B**). EC prediction using minimum intensity of the current obtained for all the electrodes, (**C**). EC prediction using maximum intensity of the current obtained for all the electrodes and (**D**). pH prediction using minimum and maximum intensity of the current obtained for all the electrodes.

The PLS-R model was constructed using the e-tongue data as: minimum and maximum intensity of the current obtained for all the electrodes, minimum intensity of the current obtained for all the electrodes and maximum intensity of the current obtained for all the electrodes, respectively for the prediction of free acidity, pH, EC and 5-HMF. In Table 3 are presented the statistical parameters for the prediction of free acidity, EC and 5-HMF. As it can be observed from the data presented in Table 3, only the prediction of EC reached high regression coefficients for e-tongue data. In the case of pH the minimum and maximum current intensity could predict this parameters using the PLS-R but not with high precision (the regression coefficient for calibration was 0.704, while for validation 0.516). In Figure 6 is presented the correlation between the experimental data vs. predicted data for EC and pH. For EC and pH prediction, there were obtained better results when the PLS-R model was built using the minimum and maximum intensity of the current obtained for all the electrodes.

## 4. Conclusions

Based on the LDA and SVM results and taking into account that the e-tongue procedure takes little time and is consuming less solvents, this method is better for honey adulteration detection. EC and pH prediction was better when the PLS-R models were built using the minimum and maximum intensity of the current obtained for all the electrodes. By combining physicochemical parameters with e-tongue, only for the calibration step LDA accuracy, sensitivity and specificity were improved compared to the LDA results obtained for e-tongue. In the case of SMV, all statistical parameters were 100% in both the calibration and validation steps. The silver and copper electrodes provided the clearest voltammograms, from which the adulterated samples could be distinguished depending on the degree of adulteration for each adulteration agent. Regarding the physicochemical parameters, the significant changes (*p* < 0.0001) between the authentic and adulterated honey samples were in the case of free acidity and HMF content.

## Figures and Tables

**Table 1:** Physicochemical parameter for authentic and adulterated honey samples using ANOVA (mean values).

Parameter	Honey		F-Value
	Adulterated	Authentic	
Free acidity (meq/kg)	11.69 (1.28) ^a^	28.14(1.57) ^b^	66.05 ***
pH	4.29(0.04) ^a^	4.31(0.05) ^a^	0.07 ^ns^
EC (μS·cm^−1^)	328.91(18.39) ^a^	373.08(22.52) ^a^	2.31 ^ns^
5-HMF (mg/kg)	64.62(7.18) ^b^	8.94(8.79) ^a^	24.06 ***

^ns^—*p* > 0.05, ***—*p* < 0.0001. ^a,b^ statistical different gropus.

**Table 2:** Statistical parameters of the LDA and SVM discrimination of authentic and adulterated honeys.

		Accuracy (%)	Sensitivity (%)	Specificity (%)
e-Tongue				
**LDA**	**Calibration**	92.31	82.61	98.59
	**Validation**	96.55	91.67	100.00
**SVM**	**Calibration**	100.00	100.00	100.00
	**Validation**	100.00	100.00	100.00
**Physicochemical parameters**				
**LDA**	**Calibration**	84.61	61.70	100
	**Validation**	89.65	73.91	100
**SVM**	**Calibration**	100.00	100.00	100.00
	**Validation**	100.00	100.00	100.00
**Physicochemical parameters + e-tongue**				
**LDA**	**Calibration**	94.87	85.71	100
	**Validation**	89.65	78.57	100
**SVM**	**Calibration**	100.00	100.00	100.00
	**Validation**	100.00	100.00	100.00

**Table 3:** Regression parameters of the calibration and validation procedure calculated for the e-tongue data submitted to partial least square regression (PLS-R) analysis.

Parameter	Data	Calibration				Validation			
		Slope	Offset	RMSE	R^2^	Slope	Offset	RMSE	R^2^
**Free acidity**	E-tongue (mimum + maxim)	0.344	11.056	11.271	0.344	0.241	15.530	15.652	0.245
	E-tongue—minimum	0.335	11.211	11.349	0.335	0.237	15.764	15.690	0.241
	E-tongue—maximum	0.288	12.009	11.746	0.288	0.242	14.740	15.624	0.247
**pH**	E-tongue (mimum + maxim)	0.703	1.281	0.243	0.704	0.570	1.916	0.303	0.516
	E-tongue—minimum	0.506	2.138	0.314	0.506	0.496	2.207	0.331	0.424
	E-tongue—maximum	0.518	2.084	0.310	0.518	0.370	2.779	0.335	0.411
**EC**	E-tongue (mimum + maxim)	0.940	21.043	48.527	0.840	0.874	41.407	66.515	0.842
	E-tongue—minimum	0.842	55.467	78.784	0.842	0.825	52.313	77.083	0.788
	E-tongue—maximum	0.922	27.378	55.351	0.922	0.844	52.770	74.571	0.801
**HMF**	E-tongue (mimum + maxim)	0.201	33.271	72.82	0.201	0.259	16.107	59.400	0.292
	E-tongue—minimum	0.085	38.089	77.919	0.085	0.173	24.166	64.362	0.169
	E-tongue—maximum	0.193	33.604	73.188	0.193	0.275	15.900	57.671	0.333
